# Dr. Leroy McLean

**Published:** 1897-05

**Authors:** 


### Obituary.

Dr. Leroy McLean, of Troy, N. Y., died at his residence in that city April 23, 1897, aged 66 years. He received his preliminary education at the Washington Academy, Salem, N. Y., and at the Union village academy, Greenwich, N. Y. He received his medical degree at the Albany Medical College in 1855, was one year house physician at Albany hospital and for four years resident medical superintendent of Marshalls' infirmary, Troy. On the breaking out of the civil war he entered the military service and was commissioned assistant surgeon of the second regiment N. Y. Vols., with rank from May 13, 1861. He was promoted to be surgeon of the same regiment September 30, 1861, and was mustered out with his regiment May 26, 1863. He afterward reëntered the army as surgeon United States Vols., and at the close of the war returned to the practice of his profession at Troy, turning his attention almost exclusively to surgery. He went to Europe in 1866, where he spent a year in study and observation. He was a member of the Medical Society of the County of Rensselaer, of the Medical Society of the State of New York and of the American Medical Association. For many years he was attending surgeon to the Troy hospital and he attained a reputation as one of the foremost surgeons in his region of country. He was a companion of the Military Order of the Loyal Legion and a highly esteemed citizen.

